# The association of protein-bound methionine sulfoxide with proteomic basis for aging in beech seeds

**DOI:** 10.1186/s12870-024-05085-6

**Published:** 2024-05-08

**Authors:** Ewa Marzena Kalemba, Kris Gevaert, Francis Impens, Sara Dufour, Anna Czerwoniec

**Affiliations:** 1 Institute of Dendrology, Polish Academy of Sciences, Parkowa 5, Kórnik, 62-035 Poland; 2 VIB-UGent Center for Medical Biotechnology, VIB, Ghent, B-9052 Belgium; 3 Department of Biomolecular Medicine, Ghent University, Ghent, B-9052 Belgium; 4 VIB Proteomics Core, VIB, Ghent, B-9052 Belgium; 5 my3Daudio Co., Ltd, Garbary 67, Poznań, 61-001 Poland

**Keywords:** *Fagus sylvatica*, Longevity, Methionine sulfoxide, Methionine sulfoxide reductase, Oxidative stress, Proteomics, Seed storage

## Abstract

**Background:**

European beech (*Fagus sylvatica* L.) trees produce seeds irregularly; therefore, it is necessary to store beech seeds for forestation. Despite the acquisition of desiccation tolerance during development, beech seeds are classified as intermediate because they lose viability during long-term storage faster than typical orthodox seeds. In this study, beech seeds stored for short (3 years) or long (20 years) periods under optimal conditions and displaying 92 and 30% germination capacity, respectively, were compared.

**Results:**

Aged seeds displayed increased membrane damage, manifested as electrolyte leakage and lipid peroxidation levels. Analyses have been based on embryonic axes, which contained higher levels of reactive oxygen species (ROS) and higher levels of protein-bound methionine sulfoxide (MetO) in aged seeds. Using label-free quantitative proteomics, 3,949 proteins were identified, of which 2,442 were reliably quantified pointing to 24 more abundant proteins and 35 less abundant proteins in beech seeds under long-term storage conditions. Functional analyses based on gene ontology annotations revealed that nucleic acid binding activity (molecular function), ribosome organization or biogenesis and transmembrane transport (cellular processes), translational proteins (protein class) and membranous anatomical entities (cellular compartment) were affected in aged seeds. To verify whether MetO, the oxidative posttranslational modification of proteins that can be reversed via the action of methionine sulfoxide reductase (Msr) enzymes, is involved in the aging of beech seeds, we identified and quantified 226 MetO-containing proteins, among which 9 and 19 exhibited significantly up- and downregulated MetO levels, respectively, in beech seeds under long-term storage conditions. Several Msr isoforms were identified and recognized as MsrA1-like, MsrA4, MsrB5 and MsrB5-like in beech seeds. Only MsrA1-like displayed decreased abundance in aged seeds.

**Conclusions:**

We demonstrated that the loss of membrane integrity reflected in the elevated abundance of membrane proteins had a higher impact on seed aging progress than the MetO/Msr system. Proteome analyses enabled us to propose protein Sec61 and glyceraldehyde-3-phosphate dehydrogenase as potential longevity modulators in beech seeds.

**Supplementary Information:**

The online version contains supplementary material available at 10.1186/s12870-024-05085-6.

## Background

Two distinct plant seed categories exist regarding desiccation tolerance, namely, orthodox and recalcitrant seeds, with the latter being the sensitive type [1]. Indeed, orthodox seeds are suitable for long-term storage, whereas recalcitrant seeds have limited storage potential [2]. Seeds with features between these two extremes are classified as intermediate seeds [3]. Seeds of all types, including orthodox seeds, stored under optimal conditions cannot escape deterioration [4]. Seed longevity is a polygenic trait that requires the coordination of many biological processes shaping the stability of seeds in the soil and their ex situ storage time [5]. More precisely, during the seed storage protective mechanisms become less efficient, repair mechanisms become slower, and removal of toxic compounds, including reactive oxygen species (ROS), becomes less effective, resulting in the inevitable loss of seed viability [5, 6]. Recently, a genome-wide association study revealed that seed longevity is determined via two groups of genes: genes involved in ROS metabolism and detoxification, and genes involved in the development of the seed coat [7]. While ROS act as signaling molecules in the regulation of seed germination and dormancy [8], continuous accumulation of metabolic ROS contributes to seed deterioration and aging [9, 10]. Incomplete reduction of molecular oxygen (O_2_) leads to the formation of superoxide anion (O_2_^•−^), which is converted enzymatically to hydrogen peroxide (H_2_O_2_). Cellular antioxidants able to decompose H_2_O_2_ into water and oxygen are less efficient in aged tissues and H_2_O_2_ is further converted to the hydroxyl radical (^•^OH), for which no enzymatic or non-enzymatic scavenger exists. Excessive accumulation of ROS, such as H_2_O_2_, O_2_^•−^, and ^•^OH, causes triphasic deterioration in seeds of all categories: (i) slight reduction of vigor, ii) oxidative damages including lipid peroxidation and leaching of solutes, iii) damages to genetic material [11].

Oxidative damage caused by ROS is the primary factor responsible for seed aging [4]. ROS oxidize nearly all encountered molecules, including proteins [12]. Among amino acids, cysteine (Cys) and methionine (Met) are especially susceptible to oxidation, which is reversible except for hyperoxidation to sulfonic acid (Cys-SO_3_H) or sulfone (MetSO_2_) [13]. Met is converted to methionine sulfoxide (MetO), and thereby affects redox homeostasis and signaling in plants [14]. Among ROS, ^•^OH has the highest oxidative potential in MetO formation [15]. Interestingly, the ^•^OH level and MetO content were found to be correlated in desiccated *Acer platanoides* seeds categorized as orthodox, while recalcitrant sycamore seeds did not exhibit such a correlation [16]. MetO is regenerated to Met, the reduced form, via the methionine sulfoxide reductase (Msr) system, which consists of MsrA and MsrB type of enzymes [17, 18]. Msr enzymes display distinct specificity toward MetO diastereoimers. MsrA can reduce both the free and protein-bound S-diastereomer of MetO, while MsrB is specific for the protein-bound R-diastereomer [19]. The plant Msr system contains diverse A-type and B-type isoforms. For example, five MsrAs and nine MsrBs were identified in *Arabidopsis thaliana* [20], whereas the poplar genome contains five MsrAs and four MsrBs [21]. Investigating the role of MsrA4, MsrB1 and MsrB2 in the longevity of *Medicago truncatula* and *A. thaliana* seeds showed that the Msr system was less functional in deteriorated seeds [22]. Msrs are present in all plant organs, and plastidial Msrs (A4, B1 and B2) were detected in *M. truncatula* and *A. thaliana* seeds [22]. Additionally, cytosolic MsrA5 was detected *in Zea mays* seeds [23], MsrB2 and MsrB5 are upregulated in response to drought [24], and *MsrA4* was found to be involved in desiccation tolerance [25].

Global analysis of methionine oxidation has been performed predominantly in the human proteome and other non-plant systems, and revealed that proteins containing MetO exist under nonstress conditions [26], disordered proteins are highly prone to oxidation in cell extracts [27], nuclear and mitochondrial proteins are preferentially oxidized inside living cells [28], and the presence of MetO within phosphorylation motifs is a highly selective phenomenon among stress-related proteins [29]. Among global studies of MetO in plants, only *A. thaliana* catalase 2 knockout plants have been characterized in detail, revealing that proteins containing MetO display mainly oxidoreductase activity, are involved in the response to abiotic and biotic stimuli and are located primarily in chloroplasts [30]. Thus far, nothing is known about the functions of proteins containing MetO in the seed proteome in relation to aging.

Proteomic studies of seed aging have been performed in seeds of many species, including *A. thaliana* [31], *Brassica napus* [32] and *Triticum aestivum* [33]. Such studies revealed that translational capacity, mobilization of seed storage reserves, and detoxification efficiency are crucial for mechanisms associated with seed vigor [31]. Abscisic acid (ABA) is the initial factor involved in the inhibition of germination in artificially treated seeds [32], and differentially expressed proteins in aged seeds are involved in nutrient storage, enzyme activity and regulation, energy and metabolism, and responses to stimuli [33].

European beech (*Fagus sylvatica* L.) is an important tree species in forest ecosystems in Europe and is propagated mainly by seeds [34]. Seed masting occurs irregularly every 5–10 years, giving rise to a need for long-term seed storage [35, 36]. Unfortunately, beech seeds exhibit intermediate storage characteristics [37]. More precisely, beech seeds acquire desiccation tolerance during seed development [38] but display difficulties in long-term storage [39] compared to strictly orthodox seeds [37]. The accumulation of ROS in beech seeds contributes to their loss of viability [40]. Aging-related and ROS-derived damage to the root apical meristem are the main causes that beech seed germination decreases after long-term storage [41], highlighting the importance of the embryonic axes. DNA fragmentation forming the characteristic DNA ladder and indicating the onset of programmed cell death (PCD) was reported in beech seeds stored for five years and longer [41]. Irreversible oxidation of proteins, specifically carbonylation, was found a potential cause for the loss of viability of beech seeds [42]. Among reversible oxidation modifications of amino acids, reduction and oxidation reactions of protein cysteines were assumed to determine the storage capacity of beech seeds [43]. To date, proteomic analyses in *F. sylvatica* include analyses of hormone-dependent seed dormancy breaking [44], pathogen infection and wounding in roots [45], and P deficiency-induced changes in fine roots [46]. Importantly, only the last study in this list included more comprehensive gel-free, mass spectrometry-based shotgun proteomics [46].

The consequences of Met oxidation in the proteome of beech seeds remain poorly understood. Châtelain et al. [22] determined that the Msr repair system plays a decisive role in establishing and preserving seed longevity. Wojciechowska et al. [47] demonstrated that the abundance of two Msr isoforms, namely, MsrB1 and MsrB2, decreases in stored beech seeds and suggested that MsrB2 is specifically linked with the viability of beech seeds via an association with proper utilization of storage material. Other Msr isoforms have not been identified in beech seeds to date. MetO has emerged as an important oxidative posttranslational modification of proteins with no data related to seed aging. For a better understanding of how beech seeds stored in genebanks age we identified and quantified MetO in the proteome of beech seeds stored for short (3 years) or long (20 years) periods. To improve the characterization of aged beech seeds used as forest restoration material we identified proteins containing MetO. Our results provide new insights into aging mechanisms in beech seeds related to the loss of membrane integrity, changes in membrane protein abundance and dynamic changes in MetO levels in proteins associated with seed viability.

## Results

### Oxidative characteristics of seeds

**Fig. 1:**
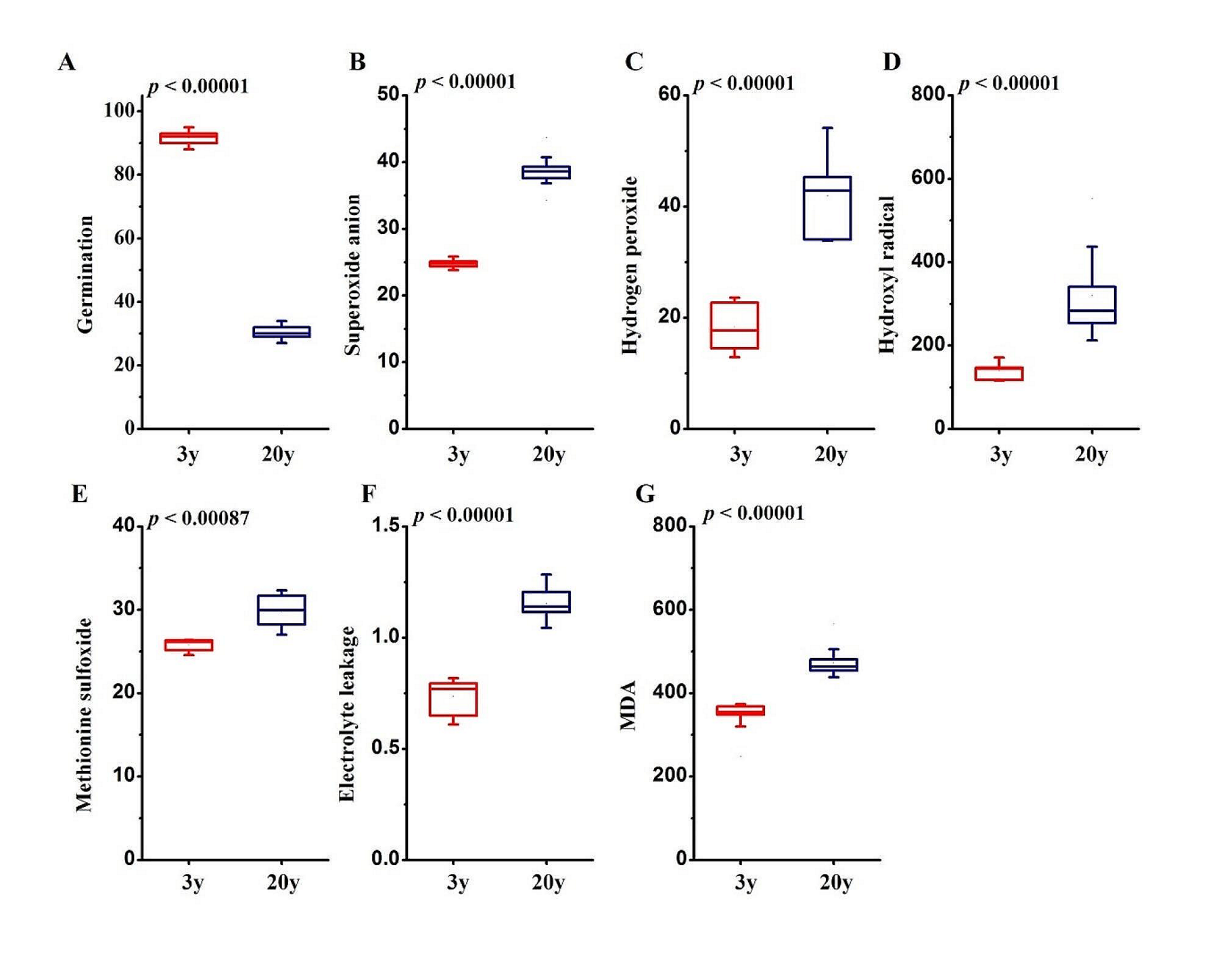
Beech seeds accumulate ROS species and damage during optimal storage. Changes in the levels of (**A**) germination capacity, (**B**) superoxide anion radical (O_2_^•−^), (**C**) hydrogen peroxide (H_2_O_2_), (**D**) hydroxyl radical (^•^OH), (**E**) methionine sulfoxide (MetO), (**F**) electrolyte leakage (EL) and (**G**) malondialdehyde (MDA) reported in embryonic axes of beech seeds under short-term (3 y) and long-term (20 y) storage. Units are expressed per gram of dry weight (DW) and per hour (h) in reactive oxygen species release determination: O_2_^•−^ [∆A_719_ g^–1^ DW h^–1^], H_2_O_2_ [pmoles g^–1^ DW h^–1^], ^•^OH [relative fluorescence units (RFU) 10^4^ g^–1^ DW h^–1^], EL [mS g^–1^ DW], MDA [nmol g^–1^ DW]. Statistical significance was confirmed with the T test

We investigated the oxidative status of beech seeds stored for short (3 years) or long (20 years) periods displaying 92 and 30% germination capacity (Fig. 1a), respectively, to verify whether they fit the oxidation-derived aging theory. Three ROS types were quantified. The levels of H_2_O_2_ and ^•^OH doubled under long-term storage, whereas the levels of O_2_^•−^ increased 1.6 times (Fig. 1b-d). The increased level of protein-bound MetO was less exacerbated compared to the ranges reported for ROS (Fig. 1e). Parameters reflecting the loss of membrane integrity and lipid peroxidation increased with age. More precisely, electrolyte leakage increased 1.6 times under long-term storage (Fig. 1f), whereas malondialdehyde (MDA) levels increased 1.4 times (Fig. 1g).

### Proteomic analysis of aging in beech seeds

**Fig. 2:**
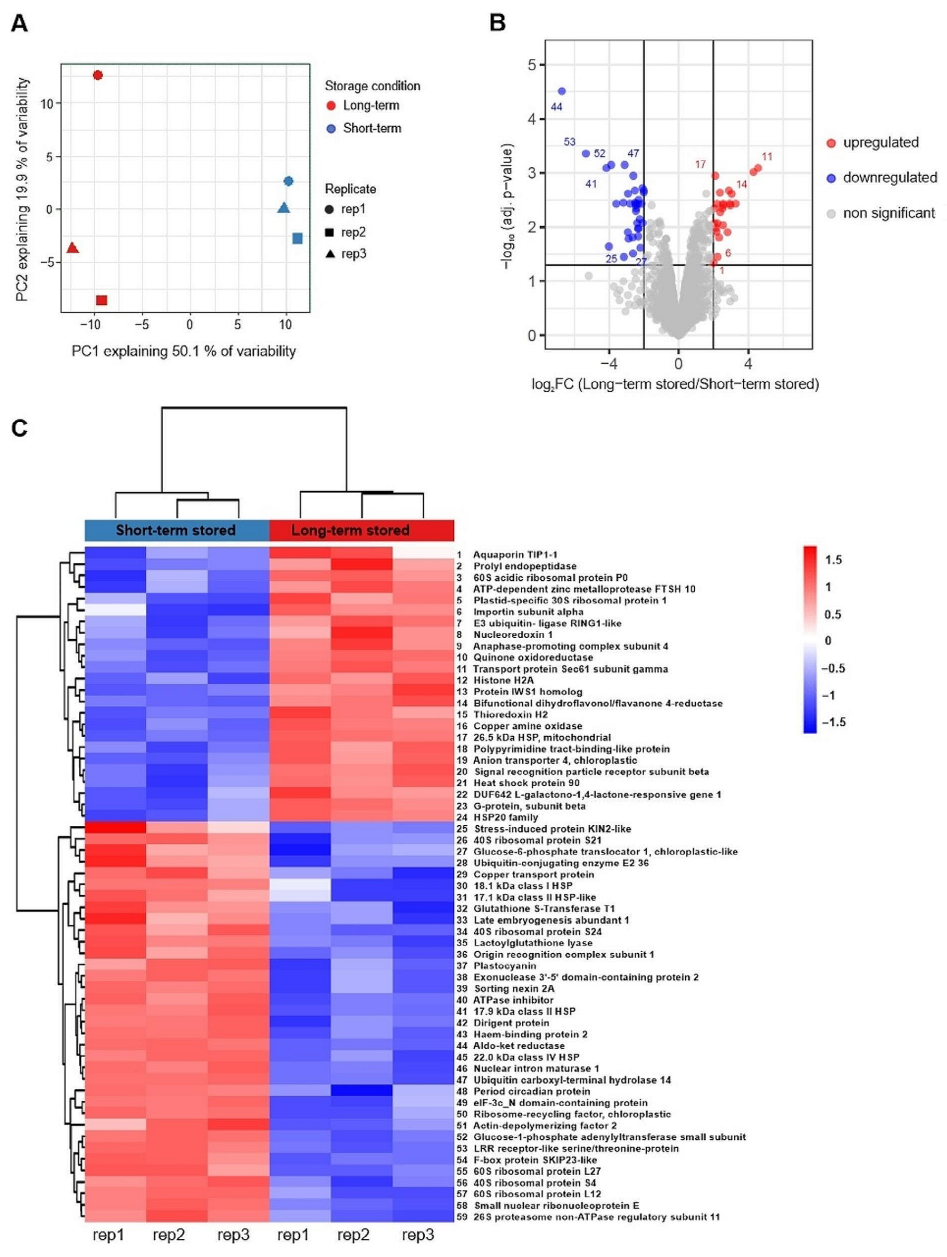
Principal component analysis performed on the replicate samples using all quantified proteins as variables (**A**) and detection of significantly regulated proteins. Volcano plot showing 24 significantly more abundant (red) and 35 significantly less abundant (blue) proteins after long term storage of beech seeds (**B**). For each protein, the fold change (in log_2_) is indicated on the X-axis while the statistical significance (in –log_10_*p*-value) is indicated on the Y-axis (FDR < 0.05 and |log_2_FC| = 2). The significantly regulated proteins were visualized in a heatmap after non-supervised hierarchical clustering of z-scored log2 LFQ intensities (**C**). Individual proteins are listed in Table S2. The color spectrum from blue to red indicates the protein expression intensity, ranging from low to high, respectively

To monitor protein changes in aging beech seeds in an untargeted manner, we compared the proteome of short-term and long-term stored seeds by label-free quantitative mass spectrometry-based proteomics. To this end, proteins from three biological replicates of each condition were extracted and digested with trypsin, after which the resulting peptide mixtures were analyzed by liquid chromatography-tandem mass spectrometry (LC-MS/MS). In total, 2,442 proteins were quantified (Table S1) with an overall similar intensity distribution in all replicate samples (Figure S1). A principal component (PC) analysis using all quantified proteins as variables clearly separated short-term and long-term stored seeds by PC1, while a wider distribution of the long-term stored seeds along PC2 indicated a larger variability among these samples (Fig. 2a). Further statistical comparison revealed 24 proteins that were significantly more abundant during long-term storage, while 35 proteins were significantly less abundant (Table S2, Fig. 2b-c).

**Fig. 3:**
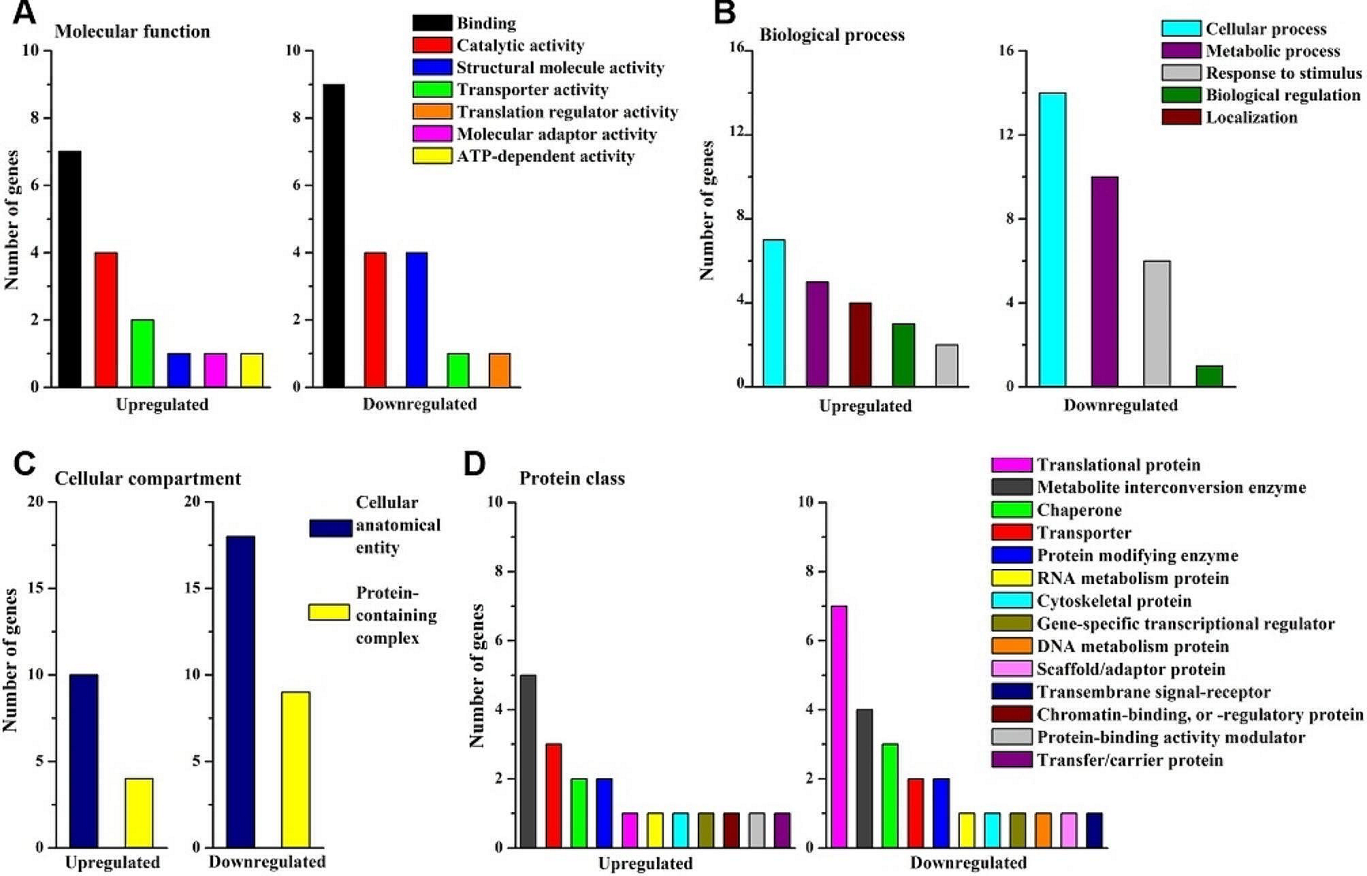
Differentially abundant proteins in long-term stored beech seeds analyzed functionally in the PANTHER knowledgebase based on protein-coding gene classification information. Gene Ontology (GO) annotations were used for classification in terms of the molecular functions (**A**), biological process (**B**), cellular component (**C**) and protein class (**D**) in groups of more abundant and less abundant proteins

Gene ontology analysis using PANTHER indicated that thirteen out of the 24 more abundant proteins were annotated as integral component of membranes being a part of the endoplasmic reticulum, vacuole, Golgi apparatus, nuclear membrane and plasma membrane (Table S2, Fig. 3). Molecular function terms based on GO revealed that binding was the most represented category both in more and less abundant proteins and consisted of heterocyclic compound binding and organic cyclic compound binding - both containing nucleic acid binding proteins, and protein binding category related to signaling receptor binding (Fig. 3A). Ion binding and amide binding were individual subclasses in more abundant proteins, whereas protein-containing complex binding subclass related to ribonucleoprotein complex binding was the characteristics of less abundant proteins. Molecular function terms also included the catalytic activity category comprising hydrolase activity, catalytic activity acting on a protein and oxidoreductase activity. Interestingly, lyase and transferase activities were recognized only in the group of less abundant proteins in aged seeds.

In the group of less abundant proteins in seeds under long-term storage, major categories of biological process included cellular process, metabolic process and response to stimulus (Fig. 3B). Cellular process category was dominated by the cellular compartment organization and biogenesis related to the ribosome organization or biogenesis, and contained subclasses unique to more abundant proteins (cellular localization, transmembrane transport) and less abundant proteins (cell cycle, actin filament-based process). Catabolic process and small molecule metabolic process (subclasses of metabolic process category) as well as response to chemical and response to endogenous stimulus (subclasses of response to stimulus category) were found only in the group of less abundant proteins. In contrast, localization category involving three subclasses (cellular localization, establishment of localization, macromolecule localization) was present only in the group of more abundant proteins.

Cellular anatomical entities involving endomembrane system, envelope, extrinsic component of membrane, intracellular anatomical structure, membrane and side of membrane were indicated in GO-based cellular component analysis of more abundant proteins in aged beech seeds (Fig. 3C).

Protein class functional analysis confirmed that among metabolite interconversion enzymes, transferases were specific to the group of less abundant proteins (Fig. 3D). Also in this group, translational protein was the predominant category in protein class terms and involved the individual subclass of translational factors. Ubiquitin-protein ligase subclass of protein modification enzyme category was also typical to the group of less abundant proteins, whereas protein binding activity modulator based on G-protein was solely reported in the group of more abundant.

### Msrs among the identified proteins

Five Msrs were identified as protein-methionine-S-oxide reductases or protein-methionine(R)-S-oxide reductases (Table 1). Further types and isoforms of Msrs might be hidden under the group of “uncharacterized proteins”, which appeared in 1891 identified records in our study. PSI-BLAST, which enables the identification of sequences with distant evolutionary relationships using position-specific scoring matrices, led to the specification of *F. sylvatica* Msr proteins as members of A1-like, A4, B5 and B-5-like isoforms (Table 1).

**Table 1:** The list of methionine sulfoxide reductases (Msrs) identified in our study with calculated changes in abundance (log_2_FC) in long-term stored seeds compared to short-term stored seeds with assigned Msr isoforms based on PSI-BLAST searches with the highest identity in amino acid sequences

Protein ID	Protein name	Gene	Identity [%]	Blasted sequence	Assigned isoform	Log_2_FC
**A-type MSRs**						
A0A2N9F608	Protein-methionine-S-oxide reductase	FSB_LOCUS10417	75	peptide methionine sulfoxide reductase A1-like (*Quercus lobate)*	MSR A1-like	**+ 1.37**
A0A2N9FJA6	Protein-methionine-S-oxide reductase	FSB_LOCUS14861	84	peptide met sulfoxide reductase 4 (*Actinidia rufa)*	MSR A4	**+ 0.53**
A0A2N9EJI3	Protein-methionine-S-oxide reductase	FSB_LOCUS10418	93	peptide methionine sulfoxide reductase A1-like (*Quercus lobate)*	MSR A1-like	**-1.62**
**B-type MSRs**						
A0A2N9F0L3	Peptide-methionine (R)-S-oxide reductase	FSB_LOCUS8291	85	peptide methionine sulfoxide reductase B5 (*Cajanus cajan)*	MSR B5	**+ 1.91**
A0A2N9IUC4	MsrB domain-containing protein	FSB_LOCUS56879	95	peptide methionine sulfoxide reductase B5-like (*Quercus suber*)	MSR B5-like	**+ 0.56**

The UniProt database contains information about two more Msrs in *F. sylvatica*, which were not reported in our study: protein-methionine-S-oxide reductase (A0A2N9GW31), for which MSR A5 is postulated because of 85% identity with the A5 isoform X1 (XP_023884431.1, *Quercus suber*), and MsrB domain-containing protein (A0A2N9I056), identified as MsrB or uncharacterized chloroplast protein and displaying low identity with AtMsrB1 and AtMsrB2, 48% and 27%, respectively.

**Fig. 4:**
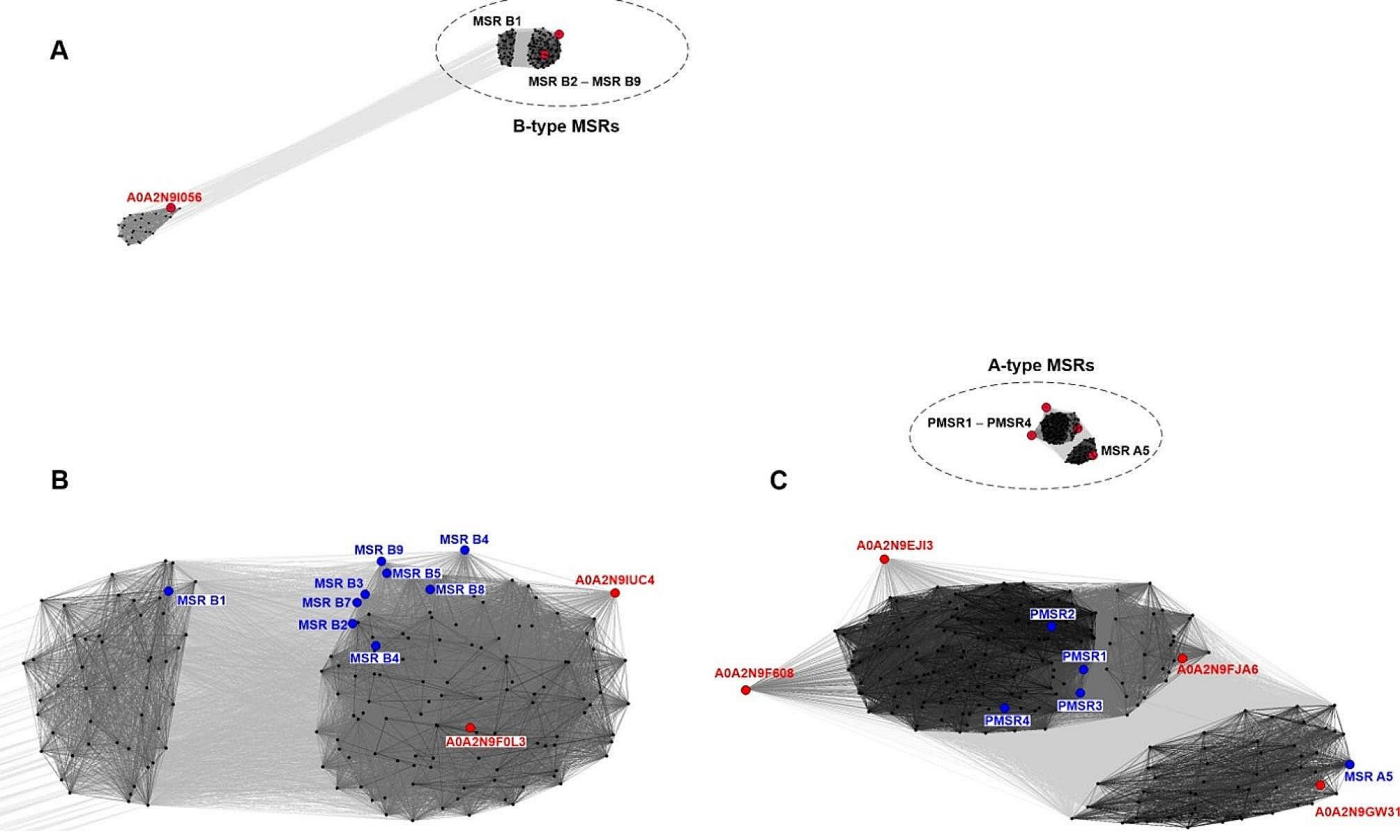
Two-dimensional visualization of protein families based on pairwise similarity performed in CLANS (CLuster ANalysis of Sequences) bioinformatics tool using protein sequences of A-type and B-type methionine sulfoxide reductases (Msrs) originating from *Arabidopsis thaliana* and *Fagus sylvatica*, and similar to the sequences identified in position specific iterations of BLAST searches (**A**). Three-dimensional graphical visualization shows relationships (lines indicate values for edges between vertices) between sequences pictured as dots. Clusters of B-type Msrs (**B**) and A-type Msrs (**C**) are enlarged to indicate the positions of Msrs from *F. sylvatica* (red dots and red accession numbers) in the network of Msrs from *A. thaliana* (blue dots and blue names). Lines indicating sequence similarity are colored by different shades of gray according to the BLAST *P* value (black: *P* value < 10^–200^, light gray: *P* value < 10^–5^)

To visualize the homology of *F. sylvatica* Msr proteins with *A. thaliana* Msrs with known properties, on which classification of MSR isoforms is based, we used the CLANS tool which revealed that beech Msrs are located on the edges of the identified clusters of Msrs (Fig. 4) and are partly similar to Arabidopsis homologs, which is in line with the *in silico* assigned isoform (Table 1).

Interestingly, pairwise similarity performed in CLANS identified a new cluster of proteins similar to A0A2N9I056 (*F. sylvatica*) not present in *A. thaliana* but displaying some homology to B1-type Msrs in this species (Fig. 4A) and containing two CysXXCys motifs at the 9–12 and 77–80 positions of amino acids in the protein sequence (Figure S2).

### MetO-containing proteins

**Fig. 5:**
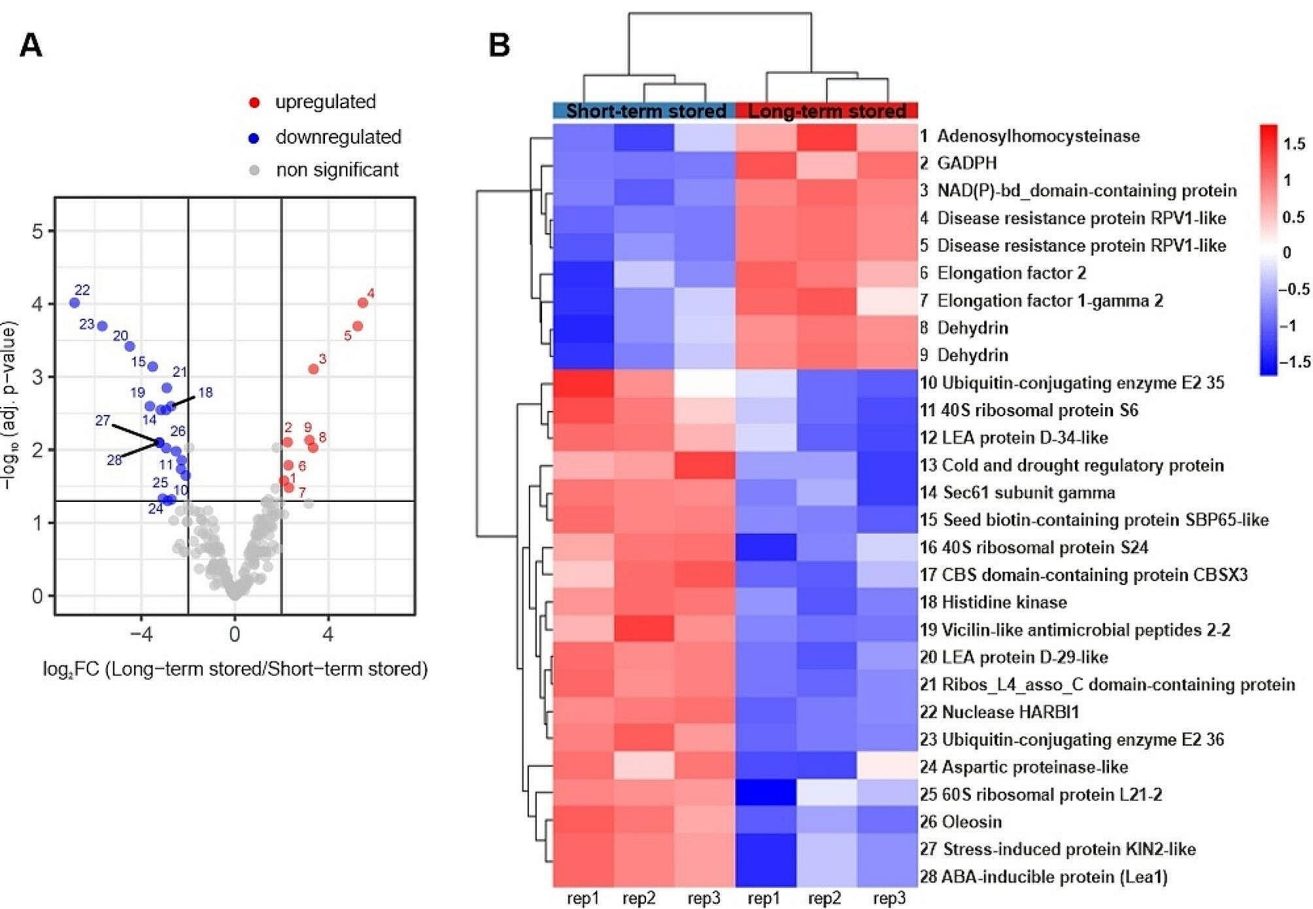
Volcano plot displaying differentially regulated methionine oxidation sites in beech seeds under short- and long-term storage (**A**). For each site, the fold change (in log_2_) is indicated on the X-axis while the statistical significance (in -log_10_*p*-value) is indicated on the Y-axis (FDR < 0.05 and |log_2_FC| = 2). The significantly regulated sites were visualized in a heatmap after non-supervised hierarchical clustering of z-scored log_2_ intensities of the MetO-containing peptides (**B**). Individual sites are listed in Table S4

In addition to protein-level changes, we also searched for oxidized methionine residues in aging beech seeds. Therefore, methionine oxidation was included as a variable modification during the spectral database searches, resulting in 226 quantified MetO-containing peptides corresponding to 221 unique methionine oxidation sites on 173 proteins (Table S3). Comparing the intensity of these peptides between short- and long-term stored seeds revealed 9 MetO sites that were significantly more abundant after long-term storage, while 19 sites were significantly less abundant (Fig. 5, Table S4).

**Fig. 6:**
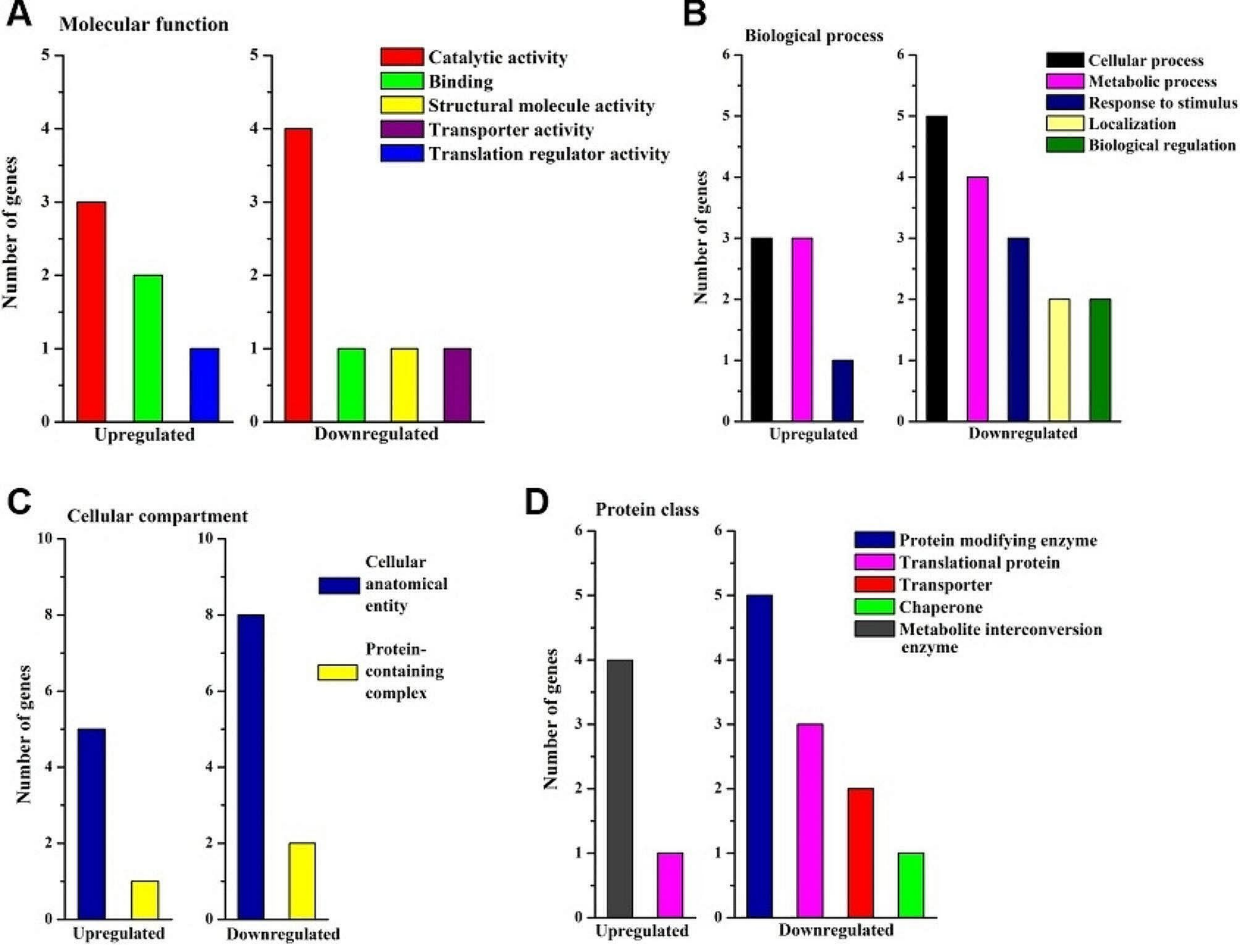
Differentially abundant proteins containing MetO sites in long-term stored beech seeds analyzed functionally in the PANTHER knowledgebase based on protein-coding gene classification information. Gene Ontology (GO) annotations were used for classification in terms of the molecular functions (**A**), biological process (**B**), cellular component (**C**) and protein class (**D**) in groups of more abundant and less abundant proteins

Molecular function terms based on GO revealed that catalytic activity was the most represented category (Fig. 6A). The oxidase subclass displayed decreasing MetO levels, whereas transferases and catalytic activity acting on proteins subclasses exhibited increasing levels of MetO. Biological process terms based on GO revealed that cellular process, metabolic process and response to stimulus were the major categories (Fig. 6B). Importantly, localization and biological regulation were two categories characteristic to the group of proteins with decreasing MetO levels. The cellular process category included cellular localization, protein folding and transmembrane subclasses, yet only in the group of proteins with decreasing MetO levels. The metabolic process category included biosynthetic process and small molecule metabolic process subclasses only in the group of proteins with increasing MetO levels. The response to stimulus category involved response to chemical and endogenous stimulus in proteins containing increasing MetO levels, and cellular response to stimulus subclass in proteins containing decreasing MetO levels.

Cellular component functional analysis demonstrated two categories among the compared groups of proteins (Fig. 6C). The cellular anatomical entity category involved five subclasses (membrane, cell periphery, endomembrane system, organelle subcompartment, perinuclear region of cytoplasm) characteristic for proteins with decreasing MetO levels. Protein containing complex category included endoplasmic reticulum protein-containing complex and membrane protein complex subclasses in the same group. No unique subclass was detected in proteins with increasing MetO levels.

Protein class functional analysis revealed that metabolite interconversion enzyme was the main category specific to the group of proteins with increasing MetO levels, whereas protein modifying enzyme was the biggest category specific to the group of proteins with less abundant MetO sites (Fig. 6D). The translational protein subclass was present in both compared protein groups, whereas transporter subclass was represented uniquely in the group of proteins with decreasing MetO levels.

## Discussion

### Beech seeds stored under optimal conditions age

Viability in seeds under long-term storage depends primarily on moisture content and storage temperature [3]. All types of seeds, including orthodox seeds, deteriorate with age [11], and autooxidation is the major determinant of aging in dry seeds [48]. We evidenced accumulation of ROS (Fig. 1b-d) and ROS-derived oxidative damage in membranes involving intensified electrolyte leakage and MDA content (Fig. 1f, g) confirming the second phase of seed deterioration [11] and demonstrating that oxidized membrane lipids are related to the viability of long-term stored seeds [4, 6, 49].

Although no specific mixture of detergents was here used to enrich the membrane protein fraction, 54% of more abundant proteins in long-term seeds were constituents of cellular membranes related to signal transduction and transport (Table S2) and involved the components of the endoplasmic reticulum membrane (i.e., translocon elements: Sec61 (A0A2N9I9C4), signal recognition particle (A0A2N9IPU2)) and nuclear envelope. Sec61 was reported as the protein with the strongest abundancy levels in seeds under long-term storage (Table S2) and having the most abundant MetO-containing peptide (Table S4), underlying its importance in the aging of beech seeds. Both, Sec61 and signal recognition particle receptor function in protein-conducting channels [50]. In this context, we hypothesize that the hydrolysis of membrane lipids causes the loss of structural integrity and contributes to the release of membrane proteins being in line with the concept of the membrane hypothesis of seed aging [51].

In this study, aged beech seeds also exhibited symptoms of the third phase of deterioration [11]. PCD is a key mechanism in seed deterioration [52] reported in seeds stored for ten years and longer [53]. To confirm this hypothesis, decreased abundance of exonuclease 3’-5’ domain-containing protein 2, which is involved in the response to DNA damage [54], was reported in aged beech seeds. The disease resistance protein (RPV1), involved in cell death signaling [55], was here found to have the highest increase in oxidation of the Met residue at two positions (Table S4). Possibly, RPV1 (A0A5C7GQD7) might be a candidate for a redox-regulated enzyme involved in the preservation of genetic material during seed storage.

### Proteomic-based indicators of aging

The identified less abundant proteins in aged beech seeds (Table S2) seem to contribute to the reduction in viability. For example, lactoylglutathione lyase, an enzyme important in the preservation of rice seed longevity and vigor [56] and aging [57], and the domain of unknown function 642 (DUF642) protein encoded by the *At3g08030* gene, a molecular marker of seed aging and germination [58]. Decreased levels of other proteins might directly cause perturbations in (i) gene expression pathways, including pre-mRNA processing (small nuclear ribonucleoprotein E (SNRPE)), ribosome assembly (RPL12, RPL27, RPS4, RPS21, RPS24), initiation of translation (eIF-3c), (ii) energy synthesis pathways, including mitochondrial complex I defects caused by nuclear intron maturase 1 [59], disruption of the oxidative pentose phosphate cycle (glucose-6-phosphate/phosphate translocator 1), (iii) DNA structure, including DNA replication course (origin recognition complex subunit 1) and double-strand break repair machinery (exonuclease 3’-5’ domain-containing protein 2), (iv) chaperones (four HSPs, Lea 1), (v) cell wall remodeling (dirigent protein), and (vi) negative regulation of the ABA signaling pathway (LRR receptor-like serine/threonine-protein and ABA inducible protein) – all (i-vi) essential for the seed germination process. However, we cannot exclude that, except ageing, the differences observed in protein levels are due to specific conditions during seed development.

#### The loss of desiccation tolerance

Proteome analyses associated with seed aging have been performed on orthodox seeds of many plant species, in which proteins displaying significant changes in abundance were involved in metabolism, protein synthesis and transport, energy, cell defense and rescue or were storage proteins [60]. Moreover, the storability of black poplar seeds, categorized as intermediate, was related to the inhibition of energy production and carbohydrate metabolism, protein turnover, and the presence of late embryogenesis abundant proteins (LEAs), and chaperonins [61]. Desiccation tolerance is associated with the accumulation of LEA proteins and small heat shock proteins (sHSP), both of which have been reported in developing beech seeds [62]. Desiccation tolerance enables long-term seed viability [63]. Therefore, the loss of desiccation tolerance was hypothesized as the reason why beech seeds do not germinate after long-term storage [42]. The levels of two HSPs were more abundant (HSP20 and HSP90-4), and four sHSPs were less abundant (HSP17.1, HSP17.9, HSP18.1, and HSP22) in seeds under long-term storage, whereas the LEA family member ABA-inducible protein (Lea1) was less abundant (Table S2). Among LEAs, the abundance of dehydrins decreased in progressively aged beech seeds, together with a decline in germination capacity [42, 64], and similar linkages were reported in Arabidopsis [31]. Both LEAs and HSPs were found to contain MetO (Table S4). An increased abundance of MetO peptides was confirmed for the dehydrin protein, while three LEA proteins (Lea1, D-34, D-29) were reported to display declining MetO levels under long-term storage. LEAs were reported to contain MetO already in mature dry (non-stored) seeds of Arabidopsis [65]. Predominantly, exposed Met residues are oxidized in properly folded and functional proteins [66], as well as intrinsically disordered proteins [27], including LEAs [67]. All oxidized Met residues identified in proteins, including several LEAs, extracted from dry Arabidopsis seeds not subjected to stress conditions, were surface exposed [65]. Disordered regions of proteins and intrinsically disordered proteins are highly prone to oxidation [27]. Kim et al. [68] assumed that Met residues are innate antioxidants or molecular bodyguards that are specifically positioned in proteins to capture ROS. Schindeldecker and Moonmann [69] proved that proteins containing MetO are structural antioxidants, particularly in mitochondria. These facts might explain why approximately 30% of protein-bound Met residues are oxidized in beech seeds (Fig. 1e).

#### Disruption of mitochondria

The search for markers of seed longevity has prompted the preservation of seed bank collections and is still in progress. One of our candidates is glyceraldehyde-3-phosphate dehydrogenase (GAPDH, A0A2N9F7Y6), a substrate of MsrB1 [70], which was found to exert increased levels of oxidation in long-term stored seeds (Table S4). Both oxidation of an exposed Met residue on GAPDH and MsrB1 depletion induce GAPDH aggregation [71]. Yoo et al. [71] suggested that MsrB1 protects GAPDH from ROS-induced oxidation of Met residues. Importantly, MsrB1 was found to decrease in stored beech seeds [47], thereby contributing to increased oxidation of Met residues in GAPDH. GAPDH aggregation causes disturbances in mitochondria and eventually oxidative stress-induced cell death [72]. Mitochondrial dysfunction was assumed to be an important determinant of the aging process in beech seeds [73]. Downregulation of levels of ATPase inhibitors (Table S2) might indicate that metabolic arrest is alleviated and rescue mechanisms are activated in aged seeds, possibly via the action of increased abundance of thioredoxin H2 (Table S2). However, the activation of pro-survival mechanisms fails in deteriorated seeds [74].

The observed elevated levels of mitochondrial ATP-dependent zinc metalloprotease FTSH10 might indicate damage of mitochondrial inner membranes, particularly as FTSH10 spans the inner mitochondrial membrane twice [75], and such damage is in line with the fact that mitochondria in aged beech seeds form aggregates and contain reduced numbers of cristae [73]. Increased levels of nuclear intron maturase 1 (NMAT1), an essential protein for mitochondrial complex I assembly and function [59], further confirm our hypothesis related to membrane damage-related aging.

#### Disturbances in translation

Rajjou et al. [31] established that translational capacity, mobilization of seed storage reserves, and detoxification efficiency are essential mechanisms for seed vigor. Indeed, 40 S ribosomal proteins (RPS4, RPS21, RPS24) as well as 60 S ribosomal proteins (RPL12, RPL27) were significantly less abundant in seeds under long-term storage (Table S2). Interestingly, 60 S acidic ribosomal protein P0 (RPLPO) is involved in the interaction between translational elongation factors. Increased RPLPO and eukaryotic translation initiation factor 3 subunit C2 (TIF32) (Table S2) indicate the readiness to initiate translation, which would not operate in ribosomes defective with many structural proteins because the disappearance of even one protein can have many implications for the disruption of normal cellular functions. Functional analyses of less abundant proteins in aged seeds indicated that the major protein class (44%) consisted of translational proteins (Fig. 3D), partly supporting this hypothesis. Perturbations in translation machinery might also occur in plastids, particularly during early stages of germination, because we showed that chloroplastic ribosome-recycling factor (RRF) was less abundant, whereas plastid-specific 30 S ribosomal protein 1 (PSRP1) was more abundant in aged beech seeds.

#### The Msr system

This study demonstrated that ^•^OH accumulated in aged beech seeds (Fig. 1d). Among ROS, ^•^OH exhibits the highest oxidation potential to generate MetO [15]. Considering MetO as a typical feature of aging in all organisms [76], the mechanism for protein repair involves enzymatic reduction of MetO via the Msr system [77]. Msr activities are correlated with seed longevity [22]. MsrB1 and MsrB2 were identified as decreasing in abundance in stored beech seeds [47]. The fact that these proteins were not identified in Table 1 might be related to the presence of 1891 “uncharacterized proteins” in the list of identified proteins (Table S1), and further types and isoforms of Msrs might be hidden under this term. Recently, MsrB5 was found to maintain vigor and longevity in rice seeds [78]. Protein A0A2N9F0L3, assigned as MsrB5, increased in abundance in aged beech seeds (Table 1).

Our research allowed the identification of A-type isoforms (A1-like and A4) of Msrs in beech seeds, however, their abundancy did not change significantly (Table 1). MsrA protects proteins from oxidative damage [79], modulate growth [80, 81] and lifespan [82]. MsrA4, functioning as a desiccation-tolerant modulator [25], prevents Met oxidation in chloroplastic HSP21, enabling its native conformation and activity [83]. Chloroplastic sHSP, a member of the HSP20 family, increased in abundance in aged beech seeds (Table S2). Additionally, the abundance of sHSP migrating in a SDS-PAGE gel as a 22 kDa protein and recognized by an antibody to 17.4 sHSP from *A. thaliana* increased as beech seeds aged [64]. No sHSP was identified to exhibit significant changes in MetO content (Table S4). These results are consistent with the increasing abundance of an A0A2N9FJA6 protein assigned as the A4 isoform (Table 1).

Beech A- and B-type Msrs and their homologs were grouped into two distinct clusters, except for the MsrB domain-containing protein (A0A2N9I056), which was separated into a distinct cluster of proteins related to the B1 isoform (Fig. 4). A0A2N9I056 contains a redox-active center, Zn^2+^ binding activity, and the biological process in which it is involved comprises response to oxidative stress [84]. Zinc-containing MsrB was assumed to be the prototypical enzyme that lost the two CysXXCys motifs later in evolution and the ancestor of B-type isoforms [85] with no homolog in Arabidopsis. In this context, elucidation of the function of A0A2N9I056 in beech seeds seems to be particularly interesting.

#### Viability-related proteins containing MetO

Global, high-throughput analyses of MetO were performed predominantly in proteomes of species with sequenced genomes [26, 27, 30]. Specific proteins that were probably not active by increasing the MetO content should be analyzed in terms of potential disruption of the biological processes in which they are involved. For example, GAPDH, above discussed potential viability indicator, whose activity is regulated by the Met redox state.

Importantly, MetO levels were less abundant in oleosin, vicilin-like antimicrobial peptides 2–2, nuclease Harbinger transposon-derived HARBI1 (nuclease HARBI1), cystathionine β-synthase X (CBSX), and aspartic proteinase. Oleosin was identified in aged wheat seeds and proposed to be regarded as a new marker of seed deterioration [33]. Antimicrobial peptides, including HARBI1, affect lifespan by preventing dysbiosis during aging [86]. HARBI1 is involved in DNA-dependent Nazaroptotic cell death, which has so far only been described in human cell lines [87]. CBSX is known to modulate the activity of thioredoxins and thereby regulate ROS homeostasis and affect plant growth and development as well as Calvin cycle enzymes, predominantly malate dehydrogenase [88], processes important in the germination process in seeds. Studies of *Arabidopsis* mutants deficient in mitochondrial malate dehydrogenase revealed that impaired seed metabolism resulted in reduced seed vigor and affected post-germination growth [89]. Aspartic proteinases are responsible for protein processing or degradation in many stages of the plant life cycle, including stress responses [90], seed maturation and germination [91]. Additionally, enzymatic elements of the ubiquitin–proteasome pathway were shown to be involved in the mitigation of stress accompanying seed germination [92]. All the above proteins affecting seed longevity and therefore the ability to germinate are under control of Met redox cycling. Interestingly, MetO-containing proteins with dynamic changes in MetO levels (40 S ribosomal proteins, elongation factors, GADPH, and dehydrins) were previously identified as carbonylated proteins in aged beech seeds with substantial changes in carbonylation levels [42]. In this context, these proteins might be assumed to be particularly sensitive to oxidation.

## Conclusions

Proteomics analysis of aged beech seeds provided evidence confirming the membrane hypothesis of aging. Protein Sec61, reported as the most abundant protein in aged seeds and the least abundancy of MetO content, is postulated to be a good candidate as a beech viability modulator, which needs further investigation in the area of transmembrane transport. Age-related dynamic changes in protein abundance were reported in proteins involved in PCD and desiccation tolerance. Importantly, GADPH is the second candidate to affect beech seed viability because of the oxidation-originated disturbances introduced by this protein in mitochondria and our proteomic evidence for damage to the mitochondrial inner membrane. We identified new Msr isoforms in beech seeds, namely, A1-like, A4, B5 and B5-like, among which MsrB5, known as a seed viability modulator from the literature, appears to be very important. Identification and quantification of MetO in proteins during beech seed aging revealed that GADPH, and probably its activity, is regulated by the Met redox state. Additionally, proteins previously proposed in the literature as seed deterioration markers or important for germination were demonstrated to be oxidized at Met residues, underlying the importance of this oxidative PTM in seed longevity.

## Materials and methods

### Plant material

Seeds of the European beech (*Fagus sylvatica* L.), originating from two provenances Kórnik (52°15′N 17°60′E) and Gryfino (53°15′N 14°29′E) in Poland, were dried to 7–8% water content and stored in closed plastic containers at optimal conditions such as no light and − 10 °C [37]. Seeds stored for 3 (Gryfino provenance) and 20 years (Kórnik provenance) displaying 92 and 30% germination capacity, respectively, were analyzed. Seed coats were removed, and the embryonic axes were isolated for the experiments. Samples consisted of three replicates of 20 axes each except for the determination of ROS release for which six replicates of ten embryonic axes were used.

### Determination of MetO levels

Determination of methionine sulfoxide (MetO) and methionine (Met) levels was performed according to the method described by Baxter et al. [93] using an Agilent Infinity II 1260 model HPLC system (Agilent Technologies, Wilmington, DE, USA) equipped with an Agilent Poroshell 120 Stablebond Aq, 3.0 × 150 mm, 2.7 μm particle column heated to 40 °C and mobile phases based on water (A) and potassium phosphate buffer (pH 2.9) combined with acetonitrile and isopropanol (B). Proteins isolated in PIPES buffer were digested using pronase, leucine aminopeptidase and prolidase for 20 h at 37 °C. MetO and Met were detected at 214 nm (reference at 590 nm) using the Agilent 1260 Infinity II Diode Array Detector. The elution program was 0% B from 0.0 to 5.0 min (flow rate of 0.15 mL min^–1^), 0 to 16% B from 5.0 to 8.0 min (flow rate of 0.3 mL min^–1^), 16 to 100% B from 8.0 to 16.0 min (flow rate of 0.3 mL min^–1^), and 0% B from 16.0 to 18.0 min (flow rate from 0.3 to 0.15 mL min^–1^). The MetO ratio was calculated in relation to the total pool of Met (MetO + Met) detected based on calibration curves. The calibration curves were made using serial dilutions of a mixture of standards (Met, MetO, Tyr and Trp, all detected as peaks at 214 nm) exhibiting identical concentration in each run.

### Determination of ROS release

#### Hydrogen peroxide

The level of H_2_O_2_ release was measured according to the method described by Schopfer et al. [94]. Reaction solution (1.2 mL) contained 20 mM phosphate buffer (pH 6), 5 μM scopoletin, and 1 U mL^− 1^ peroxidase. Seeds were incubated in darkness on a shaker at 150 rpm for 1 h at room temperature (RT). The fluorescence (λex = 346/λem = 455 nm) was measured using an Infinite M200 PRO (Tecan, Männedorf, Switzerland) plate reader and Magellan software. The results are shown in picomoles of H_2_O_2_ per gram of dry weight (DW) per hour.

#### Superoxide anion radical

The release of superoxide anion radicals was determined using a method described by Choi et al. [95]. Reaction mixture (1.2 mL) consisted of 50 mM phosphate buffer (pH 7.8), 0.05% nitro blue tetrazolium (Sigma, St. Louis, MO, USA), and 10 mM sodium azide. Seeds were incubated for 30 min at room temperature in darkness. Reaction solution (750 μL) was heated for 30 min at 85 °C, cooled and centrifuged for 1.5 min at 10 000 x RCF. The precipitate was dissolved in a solution of dimethyl sulfoxide in 2 M KOH by shaking for 30 min at 150 rpm and vortexing every 5 min. The absorbance was measured at 719 nm using an Infinite M200 PRO (Tecan, Männedorf, Switzerland) plate reader and Magellan software. The results are presented as ∆A_719_ values per gram of DW per hour.

#### Hydroxyl radical

The level of released ^•^OH was determined according to the methods of Schopfer et al. [94]. Reaction mixture (1.2 mL) contained 20 mM phosphate buffer (pH 6) and 2.5 mM sodium benzoate. Seeds were incubated for 3 h at RT in darkness on a shaker at 150 rpm. The fluorescence (λex = 305/λem = 407 nm) was measured using an Infinite M200 PRO (Tecan, Männedorf, Switzerland) plate reader and Magellan software. The results are expressed in relative fluorescence units (RFU) per gram of DW per hour.

### Electrolyte leakage

Samples were immersed for 24 h at RT in 10 mL of nanopure water (< 18.2 MΩ). The electrolytic conductivity of the solution was measured using a SevenEasy (Mettler Toledo) conductometer equipped with an InLab® 730 electrode. The results are expressed in mS g^–1^DW units.

### Malondialdehyde (MDA)

Samples were homogenized in 5% trichloroacetic acid. Lipid peroxidation was determined by the reaction of MDA with thiobarbituric acid (TBA) to form the brown red product detected colorimetrically at 532 nm. MDA content was calculated by subtracting the absorbance at 450 nm and 600 nm related to peaks generated from reactions of soluble sugars with TBA according to the method described by Agrawal and Seklani [96].

### Proteomics

A total of six samples (three referring to embryonic axes of short-term stored beech seeds and three referring to embryonic axes of long-term stored beech seeds) were prepared for LC‒MS/MS analyses. Proteins were isolated in 50 mM triethylammonium bicarbonate (TEAB) and 5% SDS buffer. Proteins (100 μg) were reduced (15 mM dithiothreitol, 30 min at 55 °C) and then alkylated (30 mM iodoacetamide, 15 min at RT in the dark). After addition of phosphoric acid (final concentration 1.2%), the samples were diluted 7-fold with binding buffer (90% methanol in 100 mM TEAB, pH 7.55). The samples were loaded on a 96-well S-Trap™ plate (Protifi), placed on top of a deepwell plate, and centrifuged for 2 min at 1,500 x g at RT. After protein binding, the S-trap™ plate was washed three times using 200 μl binding buffer and centrifuged for 2 min at 1,500 x g at RT. A new deepwell receiver plate was placed below the 96-well S-Trap™ plate, and protein digestion was performed overnight at 37 °C using 50 mM TEAB containing trypsin (1.6 μg or 1/62.5, w/w). Peptides were eluted three times: (1) with 80 μl 50 mM TEAB, (2) with 80 μl 0.2% formic acid (FA) in water and (3) with 80 μl 0.2% FA in water/acetonitrile (ACN) (50/50, v/v) using centrifugation for 2 min at 1,500 x g. The combined eluates were transferred to HPLC inserts and dried in a vacuum concentrator. Then LC‒MS/MS analysis was performed. Purified peptides were re-dissolved in 20 μl loading solvent A (0.1% TFA in water/ACN (98:2, v/v)), and the peptide concentration was determined on a Lunatic instrument (Unchained Lab). Peptides (1 μl) were injected for LC‒MS/MS analysis on an Ultimate 3000 RSLCnano system connected in-line to a Q Exactive HF BioPharma mass spectrometer (Thermo). Trapping was performed at 10 μl/min for 4 min in loading solvent A on a 20 mm trapping column (made in-house, 100 μm internal diameter (I.D.), 5 μm beads, C18 Reprosil-HD, Dr. Maisch, Germany). The peptides were separated on a 250 mm Waters nanoEase M/Z HSS T3 Column, 100 Å, 1.8 μm, 75 μm inner diameter (Waters Corporation) kept at a constant temperature of 50 °C. Peptides were eluted by a nonlinear gradient reaching 9% MS solvent B (0.1% FA in water/acetonitrile (2:8, v/v)) in 15 min, 33% MS solvent B in 100 min, 55% MS solvent B in 135 min and 97% MS solvent B in 135 min at a constant flow rate of 300 nl/min, followed by a 35-minute wash at 97% MS solvent B and re-equilibration with MS solvent A (0.1% FA in water). The mass spectrometer was operated in data-dependent mode, automatically switching between MS and MS/MS acquisition for the 16 most abundant ion peaks per MS spectrum. Full-scan MS spectra (375–1500 m/z) were acquired at a resolution of 60,000 in the Orbitrap analyzer after accumulation to a target value of 3,000,000. The 16 most intense ions above a threshold value of 13,000 were isolated with a width of 1.5 m/z for fragmentation at a normalized collision energy of 28% after filling the trap at a target value of 100,000 for a maximum of 80 ms. MS/MS spectra (200–2000 m/z) were acquired at a resolution of 15,000 in the Orbitrap analyzer. QCloud was used to control instrument longitudinal performance during the project [97].

### Data analysis

Mass spectrometry data analysis was performed in MaxQuant (version 2.1.4.0) using mainly default search settings and a false discovery rate (FDR) set at 1% at the PSM, peptide and protein levels. Spectra were searched against the *Acer* (maple trees, taxid: 4022) protein sequences in the UniProt database (database release version of February 2021) containing 33,470 sequences (https://uniprot.org) and *Fagus sylvatica* (Beechnut, taxid: 28930) protein sequences in the UniProt database (database release version of March 2021) containing 59,539 sequences [84]. Settings of the main search included: the mass tolerance for precursor (4.5 ppm); the mass tolerance for fragment ions (20 ppm); enzyme specificity (C-terminal to arginine and lysine, also allowing cleavage at proline bonds with a maximum of two missed cleavages); fixed modification (cysteine carbamidomethylation); variable modifications (oxidation of methionine residues and acetylation of protein N-termini). A matching time window (0.7 min) and an alignment time window (20 min) were used for matching between runs. Only proteins with at least one unique or razor peptide were retained. The MaxLFQ algorithm was used for protein quantification with a minimum ratio count of two unique or razor peptides. A total of 263,180 peptide-to-spectrum matches (PSMs) were performed, resulting in 29,512 identified peptides, corresponding to 3,949 identified proteins. An in-house R script using the proteinGroups output table from MaxQuant was applied for data analysis of the shotgun results. Reverse database hits were removed, LFQ intensities were log2 transformed, and replicate samples were grouped. Proteins with less than three valid values in at least one group were removed, and missing values were imputed from a normal distribution centered around the detection limit (package DEP) [98], leading to a list of 2,442 quantified proteins in the experiment. Protein abundance between pairs of sample groups (short-term stored vs. long-term stored sample groups) was compared. Statistical testing for differences between two group means was performed using the limma package [99]. Statistical significance for differential regulation was set to FDR of < 0.05 and fold change (FC) of > 4- or < 0.25-fold (|log_2_FC| = 2). The results are presented in Supplementary Tables S1 and S3. Z scored LFQ intensities from significantly up- and downregulated proteins were plotted in a heatmap after nonsupervised hierarchical clustering.

Functional analysis of less abundant and more abundant proteins was performed using the Protein ANalysis THrough Evolutionary Relationships (PANTHER) knowledgebase based on protein-coding gene classification information, namely, gene ontology (GO) annotations. A list of gene identification numbers (ID) corresponding to identified proteins (Table S2) was investigated in molecular function, biological process, cellular compartment and protein class categories [100]. All of the IDs were derived from the UniProt database [84], specifically from the *A. thaliana* genome, because the gene names of *F. sylvatica* are not recognized automatically because of the lack of the whole-genome sequence.

### Posttranslational modification (PTM) analysis

Further data analysis of the MetO-containing peptides was performed with an in-house R script, using the Oxidation (M) sites output table from MaxQuant. Reverse database hits were removed the site table was expanded, the intensity values were log2 transformed, the median was subtracted and replicate samples were grouped. MetO-containing peptides with less than three valid values in at least one group were removed and missing values were imputed from a normal distribution centered around the detection limit (package DEP) [98], leading to a list of 226 quantified methionine oxidized peptides in the experiment, used for further data analysis. To compare methionine oxidized peptides abundance between pairs of sample groups (Short-term stored vs. Long-term stored sample groups), statistical testing for differences between two group means was performed, using the package limma as described above for the shotgun data.

### Bioinformatics analyses

Proteins identified in our study as methionine reductases were assigned to the specific isoform by using Position-Specific Iterated Basic Local Alignment Search Tool (PSI-BLAST), which is the best method of searching for functional homologs of a protein [101]. Then, specific isoforms were indicated based on the highest score of sequence identity after repeated iterations.

Classification of Msr isoforms is based on *A. thaliana* proteins. Therefore, all accessible databases were searched for peptide-methionine (R)-S-oxide reductases originating from *Fagus sylvatica*. Each of six *F. sylvatica* sequences as well as A-type and B-type Msrs originating from *A. thaliana* were used as a query in a separate PSI-BLAST search. All collections of closely related sequences were used to perform multiple sequence alignment (MSA) using the MUltiple Sequence Comparison by Log-Expectation (MUSCLE) algorithm (https://www.ebi.ac.uk/Tools/msa/muscle/). MSA was corrected in BioEdit 7.2 (https://bioedit.software.informer.com/7.2/) to delete redundant sequences. Visualization of protein families based on pairwise similarity was performed in the CLuster ANalysis of Sequences (CLANS) tool based on a similarity matrix obtained from the PSI-BLAST algorithm [102].

### Electronic supplementary material

Below is the link to the electronic supplementary material.

**Supplementary Material 1: Additional file 1: Figure S1**: Protein intensity distribution. Distribution of the log_2_ LFQ intensity values in each biological replicate sample referring to long-term and short-term stored beech seeds

**Supplementary Material 2: Additional file 2: Figure S2**: The amino acid sequence of A0A2N9I056 protein

**Supplementary Material 3: Additional file 3: Table S1**: The list of identified proteins in beech seeds during storage. Statistical calculations of log_2_ LFQ intensities of samples referring to short- and long-term stored seeds

**Supplementary Material 4: Additional file 4: Table S2**: The list of proteins identified in our study with calculated changes in abundance assigned as more abundant (log_2_FC > 2) and less abundant (log_2_FC<–2) in long-term stored seeds as compared to short-term stored seeds

**Supplementary Material 5: Additional file 5: Table S3**: The list of identified peptides containing oxidized methionine and respective proteins in beech seeds during storage. Statistical calculations of log_2_ LFQ intensities of samples referring to short- and long-term stored seeds

**Supplementary Material 6: Additional file 6: Table S4**: The list of identified proteins containing methionine sulfoxide (MetO) in our study with calculated changes in abundance assigned as more abundant (log_2_FC > 2) and less abundant (log_2_FC<–2) in long-term stored seeds as compared to short-term stored seeds

## Data Availability

The mass spectrometry proteomics data have been deposited to the ProteomeXchange Consortium via the PRIDE partner repository with the dataset identifier PXD046531 and 10.6019/PXD046531.

## Abbreviations

ABA: Abscisic acid
CBSX: Cystathionine β-synthase X
CLANS: CLuster ANalysis of Sequences
Cys: Cysteine
Cys-SO_3_H: Sulfonic acid
DW: Dry weight
EL: Electrolyte leakage
GAPDH: Glyceraldehyde-3-phosphate dehydrogenase
GO: Gene onthology
H_2_O_2_: Hydrogen peroxide
HARBI1: Harbinger transposon-derived HARBI1 nuclease
LC-MS/MS: Liquid chromatography-tandem mass spectrometry
LEAs: Late embryogenesis abundant proteins
MDA: Malondialdehyde
Met: Methionine
MetO: Methionine sulfoxide
MetSO_2_: Sulfone
Msr: Methionine sulfoxide reductase
NMAT1: Nuclear intron maturase 1
O_2_^•−^: Superoxide radicals
^•^OH: Hydroxyl radicals
PC: Principle component
PSI-BLAST: Position-Specific Iterated Basic Local Alignment Search Tool
PSRP1: Plastid-specific 30 S ribosomal protein 1
PTM: Posttranslational modification
ROS: Reactive oxygen species
RPLPO: 60 S acidic ribosomal protein P0
RPV1: Disease resistance protein
RRF: Ribosome-recycling factor
sHSP: Small heat shock proteins
TBA: Thiobarbituric acid
TEAB: Triethylammonium bicarbonate
TIF32: Eukaryotic translation initiation factor 3 subunit C2
