# Supplementary material for: The association of protein-bound methionine sulfoxide with proteomic basis for aging in beech seeds
Source: BMC Plant Biol. 2024 May 8;24:377. doi: 10.1186/s12870-024-05085-6 (PMC11077735; doi:10.1186/s12870-024-05085-6)

**Figure S1. Protein intensity distribution.** Distribution of the log_2_ LFQ intensity values in each biological replicate sample referring to long-term and short-term stored beech seeds.


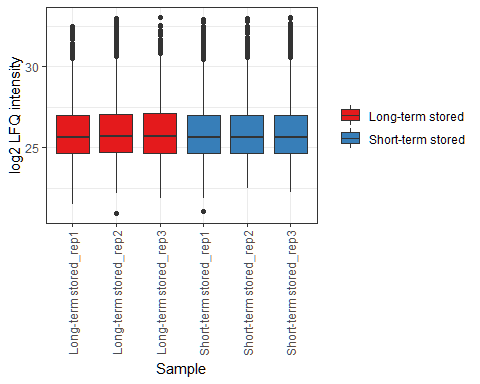

Supplement: Supplementary file 1 — Supplementary Material 1: Additional file 1: Figure S1: Protein intensity distribution. Distribution of the log2 LFQ intensity values in each biological replicate sample referring to long-term and short-term stored beech seeds [file 12870_2024_5085_MOESM1_ESM.docx]
