# Supplementary material for: The association of protein-bound methionine sulfoxide with proteomic basis for aging in beech seeds
Source: BMC Plant Biol. 2024 May 8;24:377. doi: 10.1186/s12870-024-05085-6 (PMC11077735; doi:10.1186/s12870-024-05085-6)
Supplement: Supplementary file 2 — Supplementary Material 2: Additional file 2: Figure S2: The amino acid sequence of A0A2N9I056 protein [file 12870_2024_5085_MOESM2_ESM.docx]

**Figure S2.** The amino acid sequence of a protein (A0A2N9I056) originating from *Fagus sylvatica*. This protein is grouped into a cluster of proteins displaying some homology to B1-type Msrs and not present in *A. thaliana.* The important two CysXXCys motifs at the 9-12 and 77-80 positions of amino acids are indicated with color font and underlining.

**1 11 21 31 41**

MASSSIYS**CT** **EC**GTNLNLSA AQLYPSDFYF EAGNKGTLSF SWVDTSKFRF

**51 61 71 81 91**

EKEDKIRPFF ETLNYWGIQR KRTKII**CNSC** GRVVGHVYDD GPPLTNSAGQ

**101 111 121**

FNMGPSQVIP RAPRYRFKTK ALQITS
