# Supplementary material for: The association of protein-bound methionine sulfoxide with proteomic basis for aging in beech seeds
Source: BMC Plant Biol. 2024 May 8;24:377. doi: 10.1186/s12870-024-05085-6 (PMC11077735; doi:10.1186/s12870-024-05085-6)
Supplement: Supplementary file 4 — Supplementary Material 4: Additional file 4: Table S2: The list of proteins identified in our study with calculated changes in abundance assigned as more abundant (log2FC > 2) and less abundant (log2FC<–2) in long-term stored seeds as compared to short-term stored seeds [file 12870_2024_5085_MOESM4_ESM.docx]

**Table S2.** The list of proteins identified in our study with calculated changes in abundance assigned as more abundant (log_2_FC>2) and less abundant (log_2_FC<–2) in long-term stored seeds as compared to short-term stored seeds. *Data derived from UniProt database (UniProt Consortium 2021). Gene abbreviation refers to homological *Arabidopsis thaliana* gene recognized by protein-coding gene classification information knowledgebases. **Amino acid sequences of proteins assigned as uncharacterized, containing a specific domain or identified only to class were extracted from UniProt database and were explicated using Psi-BLAST search method (Bhagwat and Aravind 2007).

| **logFC** | **adj P val** | **Protein IDs** | **Protein names** | **Cellular localization*** | **Explicated protein name**** | **Gene names** | **Gene*** |
| --- | --- | --- | --- | --- | --- | --- | --- |
| 4.56 | 0.00081 | A0A2N9I9C4 | Transport protein Sec61 subunit gamma | ER transporter | Transport protein Sec61 subunit gamma | FSB_LOCUS50449 | At5g50460 |
| 4.29 | 0.00096 | A0A2N9IHL9 | **NAD-dependent epimerase/dehydratase domain-containing protein** | Extrinsic component of endoplasmic reticulum membrane | Bifunctional dihydroflavonol 4-reductase/flavanone 4-reductase | FSB_LOCUS51425 | At5g42800 |
| 3.27 | 0.0037 | A0A2N9IPU2 | Signal recognition particle receptor subunit beta | ER integral membrane component |  | FSB_LOCUS54410 | At5g05670 |
| 3.04 | 0.0024 | A0A2N9HQX0 | **Major facilitator superfamily (MFS) profile domain-containing protein** | Multi-pass membrane protein | Anion transporter 4, chloroplastic | FSB_LOCUS44388, EZV62_002836 | At3g46980 |
| 2.96 | 0.004 | A0A2N9EII0, A0A2N9FZF7 | Uncharacterized protein | Nuclear envelope | Guanine nucleotide-binding protein, beta subunit | FSB_LOCUS2454, FSB_LOCUS20540 | At4g34460 |
| 2.93 | 0.0037 | A0A2N9EAU0 | Uncharacterized protein | Integral component of membrane | Polypyrimidine tract-binding-like protein | FSB_LOCUS3958 | At5g53180 |
| 2.88 | 0.0021 | A0A2N9INN6 | Copper amine oxidase | Integral component of membrane |  | FSB_LOCUS55048 | At1g62810 |
| 2.8 | 0.012 | A0A2N9G2J5 | **DUF642 domain-containing protein** | Cell wall | Protein DUF642 L-GALACTONO-1,4-LACTONE-RESPONSIVE GENE 1 | FSB_LOCUS21431 | At1g80240 |
| 2.56 | 0.0037 | A0A2N9I1K3, A0A2N9IW69 | **Anaphase-promoting complex subunit 4 WD40 domain-containing protein** | Nucleolus |  | FSB_LOCUS46087, FSB_LOCUS56275 | At1g71840 |
| 2.55 | 0.004 | A0A2N9FS03 | Histone H2A | Nucleus |  | FSB_LOCUS17406 | At3g54560 |
| 2.54 | 0.0092 | A0A2N9HNT4 | 60S acidic ribosomal protein P0 | Ribosome |  | FSB_LOCUS43718 | At3g09200 |
| 2.51 | 0.0045 | A0A2N9I8R2 | SHSP domain-containing protein | Chloroplastic | HSP20 family | FSB_LOCUS48934 | At4g16555 |
| 2.39 | 0.0053 | A0A5C7IFK6 | **Histidine kinase/HSP90-like ATPase domain-containing protein** | Plant-type vacuole, plasma membrane, plant-type cell wall, plastid, Golgi apparatus, cytosol | Heat shock protein 90 | EZV62_009352 | At5g56000 |
| 2.37 | 0.0023 | A0A2N9GST3 | Quinone oxidoreductase | Cytosol/nucleus |  | FSB_LOCUS30292 | At5g54500 |
| 2.32 | 0.016 | A0A2N9INT6 | Nucleoredoxin 1 | Cytosol/nucleus | Probable thiol-disulfide oxidoreductase that may participate in various redox reactions. | FSB_LOCUS54384 | At1g60420 |
| 2.23 | 0.036 | A0A5C7I868 | Importin subunit alpha | Nucleolar membrane |  | EZV62_006533,EZV62_006465 | At3g06720 |
| 2.22 | 0.0084 | A0A2N9HU25, A0A5C7HNI1, A0A5C7HQM3, | AAA+ ATPase domain-containing protein, WRKY domain-containing protein, Peptidase_M41 domain-containing protein, | Integral component of membrane in cytoplasm | ATP-dependent zinc metalloprotease FTSH 10, mitochondrial-like | FSB_LOCUS43420, EZV62_002689, FSB_LOCUS5506 | At3g27120  At1g62300  At2g30950 |
| 2.2 | 0.0037 | A0A2N9E7L3 | Protein IWS1 homolog | Nucleus |  | FSB_LOCUS2839 | At1g32130 |
| 2.17 | 0.012 | A0A2N9GDL7 | Prolyl endopeptidase | Cytoskeleton, cytosol |  | FSB_LOCUS25594, EZV62_018739 | At5g66960 |
| 2.14 | 0.01 | A0A2N9IVU7 | **Sigma 54 modulation/S30EA ribosomal protein C-terminal domain-containing protein** | Cytosolic and plastidial small ribosomal subunit | Plastid-specific 30S ribosomal protein 1 | FSB_LOCUS56222 | At3g52150 |
| 2.1 | 0.0011 | A0A2N9J8B5 | SHSP domain-containing protein | Mitochondrion | 26.5 kDa heat shock protein, mitochondrial | FSB_LOCUS60662 | At1g52560 |
| 2.06 | 0.0045 | A0A2N9IBX7 | Thioredoxin domain-containing protein | Cytosol, mitochondrion | Thioredoxin H2 | FSB_LOCUS50114 | At5g39950 |
| 2.05 | 0.0084 | A0A2N9FC41 | Calcium-binding EF-hand | Plasma membrane, extracellular region | E3 ubiquitin- ligase RING1-like | FSB_LOCUS12236 | At5g10380 |
| 2.01 | 0.046 | A0A2N9G951 | Aquaporin TIP1-1 | Integral component of membrane, vacuole |  | FSB_LOCUS23994 | At2g36830 |
| –2 | 0.0021 | A0A2N9HJL9 | **SGTA homodimerisation domain-containing protein** | Nuclear envelope, mitochondrion | Nuclear intron maturase 1 | FSB_LOCUS40007 | At3g19070 |
| –2.02 | 0.0023 | A0A2N9J159 | **SOUL heme-binding protein** | Chloroplast thylakoid membrane | Haem-binding protein 2 | FSB_LOCUS58051 | At2g37970 |
| –2.07 | 0.0083 | A0A2N9FLT7 | Plastocyanin | Chloroplast thylakoid membrane |  | FSB_LOCUS15942 | At1g20340 |
| –2.08 | 0.0019 | A0A2N9I2L1, A0A2N9FBY1, A0A2N9HNC4, A0A2N9J778 | DUF295 domain-containing protein, DUF295 domain-containing protein, DUF295 domain-containing protein, DUF295 domain-containing protein | Cytosol, cytoskeleton, mitochondrion, phragmoplast | F-box protein SKIP23-like | FSB_LOCUS48158, FSB_LOCUS12176, FSB_LOCUS41093, FSB_LOCUS60590 | At2g17030 |
| –2.13 | 0.0037 | A0A5C7I4D3 | 40S ribosomal protein S4 | Ribosome |  | EZV62_010420 | At2g17360 |
| –2.2 | 0.024 | A0A2N9HA89 | SHSP domain-containing protein | Cytoplasm | 17.1 kDa class II heat shock protein-like | FSB_LOCUS39178 | At5g12030 |
| –2.24 | 0.0072 | A0A5C7H9F0, A0A5C7IEG3 | Eukaryotic translation initiation factor 3 subunit C, eIF-3c_N domain-containing protein | Cytosol, ribosome |  | EZV62_022558, EZV62_008071 | At3g56150 |
| –2.31 | 0.0031 | A0A2N9J910 | 60S ribosomal protein L27 | Ribosome |  | FSB_LOCUS60773 | At4g15000 |
| –2.32 | 0.011 | A0A2N9HL33 | HMA domain-containing protein | Plasma membrane | Copper transport protein | FSB_LOCUS42769 | At1g66240 |
| –2.33 | 0.0037 | A0A2N9FPY0 | Ribosomal_L12 domain-containing protein | Ribosome | 60S ribosomal protein L12 | FSB_LOCUS20659 | At2g37190 |
| –2.33 | 0.015 | A0A5C7HD30 | UBC core domain-containing protein | Nucleus | Ubiquitin-conjugating enzyme E2 36 | EZV62_020299 | At1g23260 |
| –2.34 | 0.011 | A0A5C7HPA7 | ADF-H domain-containing protein | Cytosol, cytoskeleton, mitochondrion | Actin-depolymerizing factor 2 | EZV62_016424 | At3g46010 |
| –2.38 | 0.0084 | A0A2N9EZU1,A0A2N9I623 | 40S ribosomal protein S21 | Ribosome |  | FSB_LOCUS8260, FSB_LOCUS49349 | At3g53890 |
| –2.44 | 0.0045 | A0A2N9GWR6, A0A2N9EIL2 | 3'-5' exonuclease domain-containing protein, KH domain-containing protein | Cytoplasm, integral component of membrane, intermediate filament cytoskeleton, mitochondrial matrix, mitochondrial outer membrane, mitochondrion, nucleus | Exonuclease 3'-5' domain-containing protein 2 | FSB_LOCUS31646, EZV62_013314, FSB_LOCUS2494 | At5g06450 |
| –2.46 | 0.0037 | A0A2N9GXJ7 | Glyoxalase I | Chloroplast stroma, mitochondrion | Lactoylglutathione lyase | FSB_LOCUS34928 | At1g11840 |
| –2.46 | 0.0051 | A0A2N9J729 | PX domain-containing protein | Golgi apparatus, membrane, multivesicular body membrane, retromer complex | Sorting nexin 2A | FSB_LOCUS60297 | At5g58440 |
| –2.47 | 0.004 | A0A5C7HBE7 | PCI domain-containing protein | Cytosol, nucleus, plasma membrane, proteasome complex | 26S proteasome non-ATPase regulatory subunit 11 | EZV62_019118 | At1g29150 |
| –2.51 | 0.0035 | A0A2N9ENB3 | Dirigent protein | Apoplast, cell membrane, cell wall, chloroplasts |  | FSB_LOCUS4165 | At5g42510 |
| –2.52 | 0.0021 | A0A2N9IAB5, A0A2N9FVY7 | ATPase inhibitor, Cupin type-1 domain-containing protein | Mitochondrion, mitochondrial respiratory chain complex I, extracellular secretion |  | FSB_LOCUS32990, FSB_LOCUS19177 | At5g04750 |
| –2.61 | 0.0011 | A0A2N9ELL8, A0A2N9IK25 | Sm protein E, Small nuclear ribonucleoprotein E | Nucleus |  | FSB_LOCUS3557, FSB_LOCUS53898 | At2g18740 |
| –2.62 | 0.031 | A0A2N9I9B8, A0A5C7IFI9, A0A5C7GRF4 | TPT domain-containing protein, **Sugar phosphate transporter domain-containing protein**, SWIM-type domain-containing protein | [Chloroplast membrane](https://www.ebi.ac.uk/QuickGO/term/GO:0031969) | Glucose-6-phosphate/phosphate translocator 1, chloroplastic-like | FSB_LOCUS48333, EZV62_009166, EZV62_026373 | At5g54800 |
| –2.65 | 0.016 | A0A2N9EJK7 | BTB domain-containing protein | Nucleus | Period circadian protein | FSB_LOCUS2631 | At2g21150 |
| –2.79 | 0.0037 | A0A5C7I4Z9 | Ribosome-recycling factor, chloroplastic | Chloroplast |  | EZV62_010650 | At3g63190 |
| –2.88 | 0.016 | Q9ZPA7 | ABA-inducible protein | Cytosol, plasmodesma | Late embryogenesis abundant 1 | Lea1 | At3g51810 |
| –2.91 | 0.0024 | A0A2N9J9B5, A0A2N9FPP0 | SHSP domain-containing protein | Endoplasmic reticulum | 22.0 kDa class IV heat shock protein | FSB_LOCUS61320, FSB_LOCUS16801 | At4g10250 |
| –2.92 | 0.012 | A0A2N9J100 | Glutathione S-transferase T1-like | Cytoplasm, nucleus, peroxisome, plastid | Glutathione S-Transferase T1 | FSB_LOCUS58764 | At5g41210 |
| –3.11 | 0.00071 | A0A2N9IA02 | Ubiquitin carboxyl-terminal hydrolase | Nucleus, plasmodesma | Ubiquitin carboxyl-terminal hydrolase 14 | FSB_LOCUS48866 | At3g20630 |
| –3.15 | 0.036 | A0A2N9IMM5 | Stress-induced protein KIN2-like |  |  | FSB_LOCUS53620 | At5g38760 |
| –3.18 | 0.0035 | A0A5C7I8I1 | 40S ribosomal protein S24 | Ribosome |  | EZV62_007148 | At3g04920 |
| –3.59 | 0.0037 | A0A2N9HWF4,A0A2N9F4I4, A0A2N9HL03, A0A2N9HYC8, A0A2N9HY83 | PMD domain-containing protein, DUF4283 domain-containing protein | Nucleus | Origin recognition complex subunit 1 | FSB_LOCUS44574, FSB_LOCUS9663, FSB_LOCUS40804, FSB_LOCUS46788, FSB_LOCUS44576 | At4g14700 |
| –3.88 | 0.00071 | A0A2N9FJG0 | NTP_transferase domain-containing protein | Amyloplast, chloroplast | Glucose-1-phosphate adenylyltransferase small subunit, chloroplastic/amyloplastic | FSB_LOCUS15195 | At5g48300 |
| –4.01 | 0.023 | A0A2N9ECJ3 | SHSP domain-containing protein | Cytoplasm | 18.1 kDa class I heat shock protein | FSB_LOCUS394 | At5g59720 |
| –4.16 | 0.00081 | A0A2N9HHZ5 | SHSP domain-containing protein | Cytoplasm | 17.9 kDa class II heat shock protein | FSB_LOCUS39182 | At5g12030 |
| –5.33 | 0.00044 | A0A5C7HGP1 | Protein kinase domain-containing protein | Plasma membrane, pollen tube | LRR receptor-like serine/threonine-protein | EZV62_017447 | At4g36180 |
| –6.71 | 0.000031 | A0A2N9ETF1 | **NADP-dependent oxidoreductase domain-containing protein** | Cytosol, endoplasmic reticulum | Aldo-ket reductase | FSB_LOCUS9949 | At2g37760 |
