# Supplementary material for: The association of protein-bound methionine sulfoxide with proteomic basis for aging in beech seeds
Source: BMC Plant Biol. 2024 May 8;24:377. doi: 10.1186/s12870-024-05085-6 (PMC11077735; doi:10.1186/s12870-024-05085-6)
Supplement: Supplementary file 6 — Supplementary Material 6: Additional file 6: Table S4: The list of identified proteins containing methionine sulfoxide (MetO) in our study with calculated changes in abundance assigned as more abundant (log2FC > 2) and less abundant (log2FC<–2) in long-term stored seeds as compared to short-term stored seeds [file 12870_2024_5085_MOESM6_ESM.docx]

**Table S4.** The list of proteins containing MetO identified in our study assigned as more abundant (log_2_FC>2) and less abundant (log_2_FC<–2) in imbibed Norway maple and seeds as compared to germinated sycamore seeds. Protein name was derived from UniProt database (UniProt Consortium 2021) accessed on February 2024. *Gene abbreviation refers to homological *Arabidopsis thaliana* gene recognized by protein-coding gene classification information knowledgebases. **Amino acid sequences of proteins assigned as uncharacterized, containing a specific domain or identified only to class were extracted from UniProt database and were explicated using PSI-BLAST search method (Bhagwat and Aravind 2007). Green color font of a gene refers to the chloroplastic protein.

| **Log_2_FC** | **adj P Val** | **Proteins** | **Protein names** | **Explicated protein name**** | **Gene*** | **Positions within proteins** | **Localization prob** | **PEP** | **Unique_identifier** | **Score for localization** | **Sequence window** | **Nr of Oxidation M** |
| --- | --- | --- | --- | --- | --- | --- | --- | --- | --- | --- | --- | --- |
| 3.35 | 0.0093 | A0A2N9GQ23 | Dehydrin |  | At5g66400 | 125 | 1 | 0.014482 | UID478 | 97.565 | AYYDQGQGDQQNKGLMDKVMEKIPGAGNKDK | 1, 2 |
| 3.19 | 0.0074 | A0A2N9GQ23 | Dehydrin |  | At5g66400 | 129 | 1 | 0.014482 | UID479 | 97.565 | QGQGDQQNKGLMDKVMEKIPGAGNKDKIHGD | 1, 2 |
| 2.25 | 0.0079 | A0A2N9F7Y6, A0A2N9FGF0, A0A2N9HEX3 | Glyceraldehyde-3-phosphate dehydrogenase |  | At3g04120 | 87, 132, 135 | 1 | 0.000574 | UID78 | 74.944 | GAKKVIISAPSKDAPMFVVGVNEKDYTPELD, GAKKVIISAPSKDAPMFVVGVNEKEYKPEFN, GAKKVVISAPSKDAPMFVVGVNEKEYKPELD | 1 |
| 2.31 | 0.033 | A0A2N9EMF7 | Elongation factor 1-gamma 2 |  | At1g09640 | 403 | 1 | 7.79E-06 | UID37 | 119.65 | KVDITDEDQKERVSQMIEDFEPFEGEPLLDA | 1 |
| 2.1 | 0.026 | A0A2N9IW00 | Adenosylhomocysteinase |  | At4g13940 | 357 | 1 | 8.86E-06 | UID285 | 81.055 | NAIICNIGHFDNEIDMLGLENYPGIKRITIK | 1 |
| 3.37 | 0.00078 | A0A2N9FLD9 | NAD(P)-bd_dom domain-containing protein |  | AT5g10730 | 208 | 1 | 1.48E-38 | UID104 | 53.793 | GSMKIPLGVIGSPLEMVLQYAKPLNQLPLVG | 1 |
| 5.47 | 0.000096 | A0A5C7GQD7 | C-JID domain-containing protein | Disease resistance protein RPV1-like | ADR2 | 928 | 1 | 0.017938 | UID615 | 47.752 | ETRDGHKEGFFNEFTMFCMLILPIEDRRFID | 2 |
| 5.25 | 0.0002 | A0A5C7GQD7 | C-JID domain-containing protein | Disease resistance protein RPV1-like | ADR2 | 931 | 1 | 0.017938 | UID616 | 47.752 | DGHKEGFFNEFTMFCMLILPIEDRRFIDSDH | 2 |
| 2.29 | 0.016 | A0A2N9F6I5.A0A2N9FHS7 | Tr-type G domain-containing protein | Elongation factor 2 | At1g56070 | 594, 594 | 1 | 7.96E-05 | UID77 | 102.66 | KSPNKHNRLYMEARPMEEGLAEAIDDGRIGP | 1 |

| **Log_2_FC** | **adj P Val** | **Proteins** | **Protein names** | **Explicated protein name**** | **Gene*** | **Positions within proteins** | **Localization prob** | **PEP** | **Unique_identifier** | **Score for localization** | **Sequence window** | **Number of Oxidation M** |
| --- | --- | --- | --- | --- | --- | --- | --- | --- | --- | --- | --- | --- |
| -2.91 | 0.0014 | A0A2N9IWQ7 | Ribos_L4_asso_C domain-containing protein |  | At5g02040 | 343 | 1 | 0.008887 | UID288 | 124.92 | NALLKLNPYAKTARRMALLAEADRVKSKKEK | 1 |
| -2.94 | 0.0095 | A0A2N9H0D8 | Late embryogenesis abundant protein D-34-like |  | At4g26080 | 195 | 1 | 1.29E-27 | UID191 | 105.06 | VAAMAQSAADVNPRVMLDENKTTLSDVLADA | 1 |
| -3.08 | 0.047 | A0A2N9HTU3, A0A5C7GWM8 | 60S ribosomal protein L21-2 |  | At1g57660 | 140, 114 | 1 | 3.51E-09 | UID228 | 61.25 | VISTKRQPKGPKPGFMVEGATLETVTPIPYD | 1 |
| -2.87 | 0.05 | A0A2N9J6T8 | Aspartic proteinase-like |  | At1g11910 | 339 | 1 | 0.009892 | UID295 | 159.28 | ECKAVVAEYGETIIKMILEKDQPQKICSQIG | 1 |
| -2.73 | 0.0025 | A0A2N9FR54, A0A5C7H6K2 | Histidine kinase/HSP90-like ATPase domain-containing protein |  | At5g56030 | 460, 459 | 1 | 0.002503 | UID106 | 150.27 | GLVDSDTLPLNVSREMLQQHSSLKTIKKKLI | 1 |
| -3.52 | 0.00072 | A0A2N9J469 | Seed biotin-containing protein SBP65-like |  | At2g39940 | 81 | 1 | 0.00508 | UID293 | 65.395 | EKQKEKNADQARSRNMVEDKKVEARGDRREG | 1 |
| -2.27 | 0.014 | A0A2N9I9Z0 | Cold and drought regulatory protein |  | At1g54410 | 5 | 1 | 2.09E-06 | UID258 | 132.05 | ___________MAGIMHKIEETLHLGGKKKE | 1 |
| -4.5 | 0.00038 | A0A2N9EIB6 | Late embryogenesis abundant protein D-29-like |  | At3g15670 | 139 | 1 | 2.48E-15 | UID22 | 203.7 | EAKERASQKAGETKNMATQKAEEAKEAAKNK | 1 |
| -3.64 | 0.0025 | A0A2N9FS13 | Vicilin-like antimicrobial peptides 2-2 |  | At2g28490 | 89 | 1 | 0.011985 | UID114 | 116.05 | LLQESKRVVKTDAGEMRVVRSFDGRVVDRRM | 1 |
| -2.95 | 0.0028 | A0A2N9H1P1 | Oleosin |  | At4g25140 | 14 | 1 | 4.72E-12 | UID193 | 158.05 | __MSDQSKDSKPFSQMLPESAPSSRQVAKFL | 1 |
| -5.67 | 0.0002 | A0A5C7IC75 | UBC core domain-containing protein | Ubiquitin-conjugating enzyme E2 35 | At1g78870 | 34 | 1 | 0.019584 | UID308 | 58.159 | SEPAPGISASPSEDNMRYFNVMILGPTQSPY | 1 |
| -3.17 | 0.0028 | A0A2N9I9C4 | Protein transport protein Sec61 subunit gamma |  | At4g24920 | 1 | 1 | 1.38E-10 | UID257 | 101.64 | _______________MDAIDSVVDPLREFSK | 1 |
| -2.1 | 0.023 | A0A2N9FFW1, A0A2N9I983, A0A5C7H9T7, A0A5C7HVD7 | 40S ribosomal protein S6 |  | At4g31700 | 1, 1, 1, 1 | 1 | 2.28E-05 | UID96 | 113.38 | _______________MKFNIANPTTGCQKKL | 1 |
| -2.71 | 0.048 | A0A5C7HD30 | UBC core domain-containing protein | Ubiquitin-conjugating enzyme E2 36 | At1g16890 | 34 | 1 | 0.020216 | UID302 | 57.52 | SEPAPGISASPSEENMRYFNVMILGPSQSPY | 1 |
| -2.3 | 0.018 | A0A2N9FLD1 | 40S ribosomal protein S24 |  | At3g04920 | 48 | 1 | 0.000848 | UID103 | 97.86 | RANVSKVELKEKLARMYEVKDPNVIFVFKFR | 1 |
| -2.51 | 0.01 | A0A2N9HT40 | CBS domain-containing protein | CBS domain-containing protein CBSX3. mitochondrial | At5g10860 | 46 | 1 | 1.12E-11 | UID225 | 105.77 | QPVMFSRFESVTSARMEEHGFESTTIADVLK | 1 |
| -6.86 | 0.000096 | A0A2N9ERN8 | Nuclease HARBI1 |  | At5g35695 | 7 | 1 | 0.015064 | UID43 | 54.95 | _________MTVALRMLVYGVAADSTDEYVR | 1 |
| -3.23 | 0.0079 | A0A2N9IMM5 | Stress-induced protein KIN2-like |  | At5g38760 | 1 | 1 | 2.84E-23 | UID270 | 217.62 | _______________MDKSQNTCFQAGQAKG | 1 |
| -3.23 | 0.0079 | Q9ZPA7 | ABA-inducible protein (Lea1) |  | At5g53820 | 1 | 1 | 2.84E-23 | UID316 | 126.51 | _______________MDKSQNTCFQAGQAKG | 1 |
